# Inflammatory biomarker levels over 48 weeks with dual vs triple lopinavir/ritonavir-based therapy: Substudy of a randomized trial

**DOI:** 10.1371/journal.pone.0221653

**Published:** 2019-09-06

**Authors:** Darrell H. S. Tan, Maria Jose Rolon, Maria Ines Figueroa, Omar Sued, Ana Gun, Rupert Kaul, Janet M. Raboud, Leah Szadkowski, Mark W. Hull, Sharon L. Walmsley, Pedro Cahn, the Argentinean GARDEL research network

**Affiliations:** 1 St. Michael’s Hospital Division of Infectious Diseases, Toronto, ON, Canada; 2 University Health Network Division of Infectious Diseases, Toronto, ON, Canada; 3 University of Toronto Department of Medicine, Toronto, ON, Canada; 4 Toronto General Hospital Research Institute, University Health Network, Toronto, ON, Canada; 5 Fundación Huésped, Pasaje Angel Peluffo 3932 (C1202ABB), Ciudad Autónoma de Buenos Aires, Argentina; 6 University of Toronto Department of Immunology, Toronto, ON, Canada; 7 Dalla Lana School of Public Health, University of Toronto, Toronto, ON, Canada; 8 St. Paul’s Hospital, Vancouver, BC, Canada; Azienda Ospedaliera Universitaria di Perugia, ITALY

## Abstract

**Background:**

Inflammation has been associated with increased morbidity and mortality in HIV-positive patients. We compared inflammatory biomarkers with dual therapy using lopinavir/ritonavir plus lamivudine (LPV/r+3TC) versus triple therapy using LPV/r plus two nucleoside reverse transcriptase inhibitors (LPV/r+2NRTIs) in treatment-naïve HIV-positive adults.

**Methods:**

This was a substudy among Argentinian participants in the randomized trial GARDEL. We measured hsCRP, IL-6, MCP-1, TNF, D-dimer and sCD14 from plasma collected at baseline, week 24 and week 48. Generalized estimating equations with an identity/logit link were used to model the average impact of dual versus triple therapy on each biomarker over time, controlling for baseline levels. Additional models estimated the average effect of virologic suppression on biomarker levels over time, adjusting for age, sex, and baseline CD4 count.

**Results:**

Of 191 trial participants enrolled in Argentina, 172 had baseline and follow-up measurements and were included. Median (IQR) age was 35.5 (28.5, 45) years and CD4 cell count was 310 (219, 414) cells/mm^3^. Dual therapy was not associated with significantly different biomarker levels over 48 weeks relative to triple therapy. Virologic suppression was associated with statistically significant decreases in MCP-1, TNF and D-dimer levels and an unexpected increase in sCD14 levels. No change was observed in hsCRP or the proportion of participants with undetectable IL-6 levels.

**Conclusions:**

In addition to having virologic non-inferiority, LPV/r+3TC dual therapy is generally associated with similar inflammatory biomarker levels over 48 weeks compared to LPV/r+2NRTIs triple therapy in treatment-naïve adults. Further study of dual treatment regimens is warranted.

## Introduction

Combination antiretroviral therapy (cART) for HIV infection has traditionally relied on three-drug regimens containing two nucleos(t)ide reverse transcriptase inhibitors (NRTIs) with a third drug from another class.[1, 2] However, the potential to reduce costs, avoid drug toxicities, and improve tolerability has driven increasing interest in studying two-drug regimens, particularly protease inhibitor and integrase inhibitor-based combinations.[3, 4]

GARDEL (Global AntiRetroviral Design Encompassing Lopinavir/r and Lamivudine vs LPV/r based standard therapy) was an open-label randomized controlled trial that demonstrated the virologic non-inferiority of an experimental dual therapy regimen of lopinavir/ritonavir with lamivudine (LPV/r + 3TC), compared to a standard triple therapy regimen of LPV/r with two NRTIs in treatment-naïve HIV-positive adults. At 48 weeks, 88.3% of participants in the dual therapy arm had HIV RNA <50 copies/mL, versus 83.7% of those in the triple therapy arm, giving a difference of 4.6% (95% confidence interval, -2.2 to 11.8) and meeting non-inferiority criteria.[5] An extension phase of the study further demonstrated that among those with virologic response at week 48, 90.3% of dual therapy and 84.4% of triple therapy participants maintained HIV RNA <50 copies/mL at week 96, giving a difference of 5.9% (95%CI, -2.3 to 14.1).[6]

Because HIV infection is characterized by increased levels of immune activation and systemic inflammation that are associated with morbidity and mortality, it is also important to consider the relative impact of novel treatment strategies on inflammatory biomarkers. Prior studies have found that inflammation decreases but does not normalize with the use of suppressive antiretroviral therapy,[7, 8] and that the choice of antiretroviral agents may have differential effects on the inflammatory milieu.[9] We therefore sought to compare the effects of dual versus triple therapy on a panel of inflammatory biomarkers among participants in the GARDEL trial from Argentina.

## Materials and methods

### Design

We conducted a post-hoc sub-analysis of the GARDEL study, an open-label, phase III clinical trial conducted at 19 centres in six countries. Full study details have been published elsewhere.[5] The GARDEL trial is registered with ClinicalTrials.gov, number NCT01237444. Study participants were randomized 1:1 in blocks of four, stratified by plasma viral load at screening (above or below 100,000 copies/mL), to either the dual or triple therapy group. The experimental dual therapy regimen consisted of co-formulated lopinavir/ritonavir 400/100 mg (LPV/r) plus lamivudine 150 mg (3TC) twice daily. Triple therapy consisted of co-formulated LPV/r twice daily plus an investigator-selected fixed-dose combination NRTI backbone of either abacavir/lamivudine (ABC/3TC), tenofovir/emtricitabine (TDF/FTC), tenofovir/lamivudine (TDF/3TC) or zidovudine/lamivudine (AZT/3TC). After the screening visit, participants were seen at baseline and then at weeks 4, 8, 12, 24, 36 and 48. The primary outcome of the study was virologic response, defined as having HIV RNA <50 copies/mL at week 48 (FDA ITT-exposed analysis). Plasma specimens were collected from the baseline, week 24 and week 48 visits for measurement of inflammatory biomarkers.

### Participants

HIV-1 infected adults were eligible for participation in the GARDEL trial if they were aged >18 years, antiretroviral-naïve with HIV RNA >1000 copies/mL, hepatitis B surface antigen seronegative, and in generally good medical condition. For feasibility reasons, only participants from the nine clinical trial sites in Argentina were eligible for inclusion in this sub-analysis; participants also needed to have plasma specimens available from the baseline visit and at least one of the week 24 and/or 48 visits for analysis. Baseline demographics of the 191 participants enrolled at the nine study sites in Argentina were compared to 227 participants enrolled at the ten sites in other countries (Chile, Mexico, Peru, Spain, United States) using Wilcoxon rank sum tests for continuous variables and chi-square tests for nominal variables. The baseline demographics of participants from Argentina who were excluded due to lack of follow up were compared to included patients using similar methods.

### Outcome measures

The primary outcome of this analysis was highly sensitive C-reactive protein (hsCRP), an acute phase reactant whose transcription is up-regulated by hepatocytes, smooth muscle cells and macrophages in response to inflammatory cytokines.[10] Increases in this biomarker occur in a wide variety of pathological conditions, and are independently associated with cardiovascular disease in the general population,[11, 12] and with progression to AIDS and death in HIV-positive populations.[13–15]

Secondary outcome measures included three inflammatory cytokines: interleukin-6 (IL-6), monocyte chemoattractant protein-1 (MCP-1) and tumour necrosis factor (TNF), which play important roles in leukocyte and endothelial cell activation; as well as D-dimer, a marker of thrombogenesis; and soluble CD14 (sCD14), a marker of monocyte activation. MCP-1 was of interest as it has been associated with hepatic and atherosclerotic comorbidity in people living with HIV.[16, 17] TNF has been linked to neurocognitive dysfunction and HIV pathogenesis.[18, 19] As for hsCRP, elevated IL-6 and D-dimer levels have been associated with an increased risk of all-cause mortality among HIV-positive persons.[14, 15]

### Laboratory analyses

Specimens were stored at -80°C and shipped on dry ice to the laboratory at the University of Toronto, Canada, for batched testing. Plasma concentrations (dilution factor) of hsCRP (1:2000), IL-6 (1:2), MCP-1 (1:2), TNF (1:2), D-dimer (1:5) and sCD14 (1:300) were measured by ELISA (Sekisui, Stamford CT for D-dimer; R&D Systems, Minneapolis MN for sCD14; MSD, Rockville MD for all others) according to the manufacturers’ instructions. Each sample was tested in duplicate, and the results averaged; those with coefficients of variation (CV) exceeding 30% were repeated. Samples with optical densities below the lowest detectable standard were also assigned the value of that standard. Lower limits of detection for each analyte were as follows: hsCRP (0.0139 ng/mL), IL-6 (0.3 pg/ml), MCP-1 (4.0 pg/ml), TNF (0.3 pg/ml), D-dimer (4.6 ng/mL), sCD14 (0.25 ng/mL).

### Statistical analysis

Generalized estimating equation (GEE) models using exchangeable correlation matrices and an identity or logit link as appropriate were used to evaluate the effect of dual therapy on each outcome measure.[20] Biomarker levels were treated as continuous variables (with logarithmic transformations as appropriate) in models with an identity link, or as binary variables (detectable versus undetectable) in models using a logit link. For each of the six biomarkers, the primary analysis was a multivariable model that included treatment assignment, baseline value of the relevant marker and visit (week 24 or week 48) as covariates. We did not impute missing outcome data. A variety of residual plots were examined to check model assumptions, determine the correct functional form of continuous covariates and examine goodness of fit of the models.

We also conducted a sensitivity analysis in which participants who did not have HIV RNA<50 copies/mL at week 48 were excluded. Further, to account for a potential effect of concomitant anti-inflammatory medications on biomarker levels, we conducted additional sensitivity analyses in which use of any such drug within 1–4 days of study visits (depending on the elimination half-life of the drug) was included in the model as a time-dependent covariate.

Finally, we quantified the effect of having plasma HIV RNA <50 copies/mL on each biomarker at each visit in both treatment groups combined, using GEE models adjusted for age, sex, and baseline CD4 count. These covariates were selected due to prior literature suggesting them to have potential relevance to HIV-associated inflammation.

### Calculation of detectable difference

The expected standard deviation (SD) in the primary outcome measure hsCRP was estimated at roughly 4 ug/mL based on published literature.[14] Using equations that account for correlation between repeated measures (rho, estimated at 0.5),[21] the anticipated sample size of 191 participants was expected to be able to detect a difference in hsCRP of 1.25 ug/mL between the dual therapy and triple therapy arms, assuming 80% power, a significance level of .05, and two visits per participant. This value was considered a clinically appropriate minimum detectable difference, corresponding to roughly one third of one SD, and being significantly less than the 2.12 ug/mL difference in baseline levels between HIV-positive adults who proceeded to death in the SMART trial versus matched controls from that trial.[14] Notably, the SD estimate of 4 ug/mL represents a conservative estimate, because the SD in our study sample of treatment naïve patients was considerably smaller than in the SMART trial. As a result, the minimum detectable difference in our study is likely smaller than suggested above.

## Results

Of 191 participants randomized for the GARDEL trial in Argentina, 19 had insufficient follow-up specimens for laboratory testing, leaving 172 participants in this analysis. The 191 participants from Argentina were older (36 vs. 31 years, p = 0.02), were less likely to be male (77% vs. 89%, p<0.001) and MSM (42% vs. 75%, p<0.0001), and had higher baseline HIV RNA (5.14 vs. 4.65 log10 copies/mL, p<0.0001) than participants from other countries. The 172 participants included in the analysis were similar to the 19 excluded participants in terms of age, gender, risk factor, and baseline CD4 count and HIV RNA.

Participants were mostly Hispanic/Latino heterosexual males. Median (interquartile range) age was 35.5 (28.5, 45) years, baseline CD4 count was 310 (219, 414) cells/mm^3^ and baseline log_10_ plasma HIV RNA was 5.15 (4.68, 5.59) copies/mL. The most commonly selected NRTI backbone in the triple therapy arm was AZT/3TC, at 88%. None of the participants had symptomatic AIDS at baseline. Baseline characteristics of the study population were similar in the dual and triple therapy groups, as summarized in Table 1.

**Table 1:** Participant characteristics.

Characteristic	Dual therapy (n = 91)	Triple therapy(n = 81)
Age	35 (28–46)	36 (29–41)
Male	69 (76%)	66 (81%)
Race		
Black	1 (1%)	1 (1%)
White	23 (25%)	16 (20%)
Hispanic/Latino	67 (74%)	64 (79%)
HIV Risk Factor		
Heterosexual	52 (57%)	42 (52%)
MSM	37 (41%)	37 (46%)
Other	2 (2%)	2 (2%)
Baseline viral load (log_10_ copies/mL)	5.2 (4.7–5.6)	5.1 (4.6–5.6)
Baseline CD4 (cells/mm^3^)	306 (205–400)	316 (228–421)
Active Comorbidities		
Diabetes	1 (1%)	1 (1%)
Hepatitis C (HCV RNA+)	1 (1%)	0
Clade		
B	51 (56%)	45 (56%)
C	6 (7%)	4 (5%)
DB	6 (7%)	6 (7%)
F	0	4 (4%)
FB	26 (29%)	21 (26%)
KB	2 (2%)	1 (1%)
NRTI backbone		
3TC	91 (100%)	0
AZT/3TC	0	71 (88%)
TDF/3TC	0	2 (2%)
TDF/FTC	0	5 (6%)
Switch AZT/3TC →TDF/3TC	0	3 (4%)

The proportion of participants with specimens available for laboratory analysis was 100% at baseline, 97.7% at week 24 and 94.8% at week 48. There was no missing data for baseline values, time, or treatment received. Of the 503 specimens analyzed for each biomarker, 80 (15.9%) were assigned the LLOD value for IL-6 due to having a reading below the LLOD and/or CV>30%; this biomarker was therefore analyzed as a binary outcome in the GEE models. We similarly assigned values equal to the LLOD for seven (1.4%) TNF results and two (0.4%) D-dimer results that were undetectable. None of the hsCRP, MCP-1 or sCD14 results had undetectable results.

**Fig 1:**
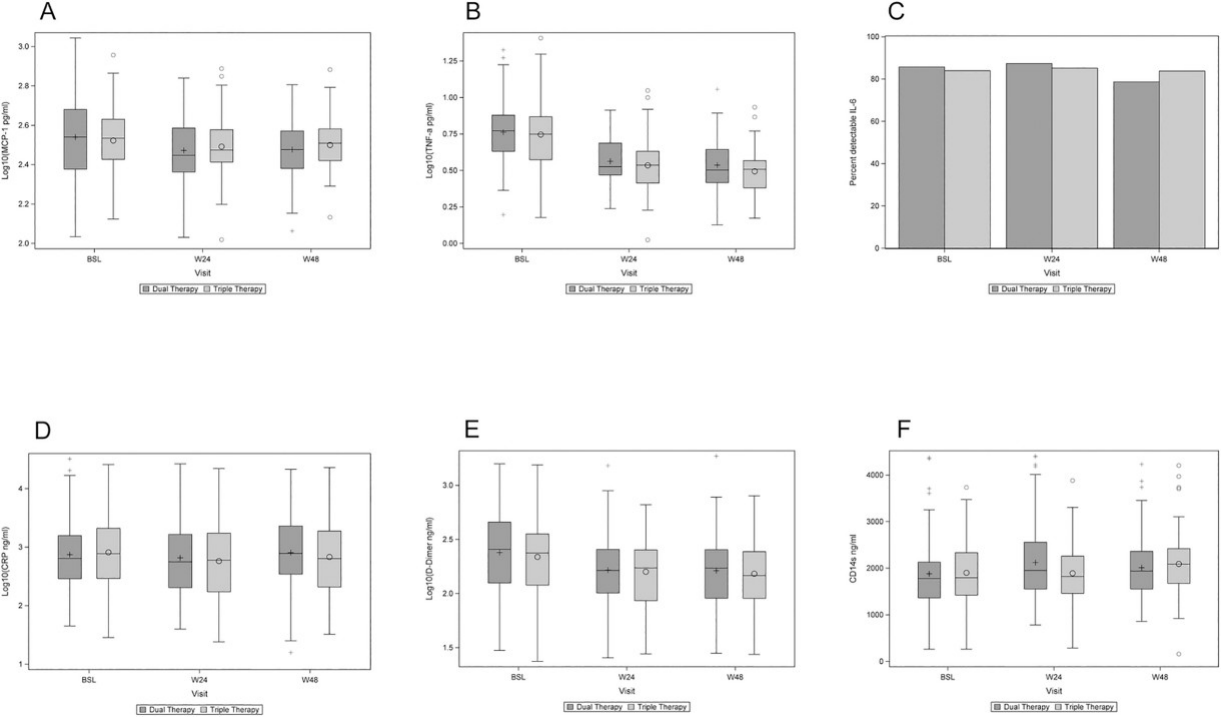
Inflammatory marker levels by study arm over 48 weeks. Boxplots showing plasma levels of a) MCP-1, b) TNF, c) IL-6, d) hsCRP, e) D-dimer and f) sCD14 by study arm over 48 weeks.

Median baseline values for each biomarker in the entire sample are shown in Table 2, along with the results of the primary analysis. The parameter estimates represent the average difference in biomarker levels over 48 weeks of follow-up in the dual therapy arm compared with the triple therapy arm. There was no statistically significant difference seen between groups for any of the biomarker levels. Fig 1 shows the distribution of biomarker levels in the two study arms at each time point.

**Table 2:** Baseline inflammatory marker levels and estimated effects associated with dual vs triple therapy from a GEE model.

Biomarker	Median baseline value (IQR)	Treatment Effect^a^	
		Estimate (95% CI)	p-value
hsCRP (log_10_ ng/mL)	2.84 (2.47–3.24)	0.09 (-0.06,0.23)	0.25
Undetectable IL-6^b^	26 (15%)	1.15 (0.59,2.25)	0.69
MCP-1 (log_10_ pg/mL)	2.53 (2.41–2.66)	-0.03 (-0.07, 0.00)	0.05
TNF (log_10_ pg/mL)	0.75 (0.62–0.88)	0.02 (-0.01,0.05)	0.18
D-dimer (log_10_ ng/mL)	2.41 (2.10–2.61)	0.00 (-0.07,0.08)	0.98
sCD14 (ng/mL)	1778 (1382–2246)	103 (-57.6,263)	0.21

^a^ From a GEE model adjusted for visit and baseline value

^b^ Values shown are the proportion having, and odds ratio of having, an undetectable value

Sensitivity analyses excluding the four participants with detectable HIV RNA at week 48 did not qualitatively impact the results. Further, 8.3% and 8.6% of participants had used an anti-inflammatory medication within five drug elimination half-lives of their week 24 and 48 visits, respectively. Sensitivity analyses in which such medication use was considered in the models as a time-dependent covariate gave qualitatively unchanged results.

To further explore the relationship between cART and systemic inflammation, we quantified the effect of having plasma HIV RNA <50 copies/mL on each outcome measure (Table 3). In GEE models adjusted for age, sex and baseline CD4 count, having an undetectable plasma viral load was associated with statistically significant decreases in MCP-1, TNF and D-dimer levels and an increase in sCD14 levels. No change was observed in hsCRP or in the proportion of participants with undetectable IL-6 levels.

**Table 3:** Results of multivariable GEE regression models estimating the effect of plasma HIV RNA <50 copies/mL on inflammatory biomarkers^a^.

Covariate	hsCRP (log_10_ ng/mL)	Undetectable IL-6^b^	MCP-1 (log_10_ pg/mL)	TNF (log_10_ pg/mL)	D-dimer (log_10_ ng/mL)	sCD14 (ng/mL)
**Undetectable viral load**	-0.04 (-0.13,0.05)	1.17 (0.73,1.88)	-0.04 (-0.06,-0.02)	-0.19 (-0.22,-0.17)	-0.12 (-0.16,-0.07)	167 (65.2,269)
**Male sex**	-0.06 (-0.26,0.14)	1.05 (0.54,2.01)	0.05 (0.01,0.10)	0.03 (-0.03,0.08)	-0.11 (-0.20,-0.02)	-131 (-315,53.3)
**Age** **(per 10 years)**	0.07 (-0.00,0.14)	0.79 (0.59,1.05)	0.02 (0.00,0.04)	0.04 (0.02,0.07)	0.08 (0.04,0.12)	78.0 (9.62,146)
**Baseline CD4 (per 100 cells/mm**^**3**^**)**	-0.01 (-0.06,0.04)	1.09 (0.91,1.29)	-0.01 (-0.02,0.00)	-0.02 (-0.03,-0.01)	-0.04 (-0.06,-0.01)	-67.8 (-107,-28.6)

^a^ Estimate (95% confidence intervals) are presented

^b^ Odds ratio (95% confidence intervals) are presented

## Discussion

In this sub-analysis of the GARDEL trial, dual therapy with LPV/r plus 3TC was associated with similar levels of inflammatory biomarkers over 48 weeks compared with LPV/r-based triple therapy in treatment-naïve HIV-positive adults. In analyses that ignored the specific treatment regimen, suppressed viral load was strongly associated with decreased levels of several inflammatory markers, including D-dimer, MCP-1 and TNF. In contrast, HIV RNA <50 copies/mL was associated with an unexpected increase in sCD14 levels, while no association was seen with IL-6 or hsCRP levels.

In evaluating the relative merits of novel treatment strategies for HIV infection, it is important to consider a range of different outcomes, including not only virologic response, immunologic recovery, toxicity and tolerability, but also changes in biomarkers of systemic inflammation. That inflammatory biomarkers are similar, rather than higher, in the setting of this dual therapy combination compared to triple therapy is reassuring, because elevated inflammatory markers have been associated with adverse outcomes. For instance, several studies have linked higher levels of hsCRP, IL-6 and D-dimer with higher risks of all-cause mortality,[14, 22–24] AIDS events,[14, 23] and major non-AIDS events.[25] These associations remain significant even after adjustment for plasma HIV RNA levels and nadir/current CD4 cell counts.

Our finding of decreases in inflammatory and coagulation biomarkers in the setting of undetectable viremia is generally consistent with prior studies among treatment-naïve patients.[26–28] Of note, however, several reports have shown differences in individual biomarkers according to cART regimen. For instance, in a substudy of the ACTG5260 trial comparing boosted atazanavir, boosted darunavir and raltegravir, each in combination with TDF/FTC, declines were generally seen in D-dimer, hsCRP and IL-6 levels, but regimen-specific differences were observed: declines in D-dimer were not seen with raltegravir, declines in hsCRP were not seen with darunavir, and declines in IL-6 were not seen with protease inhibitors.[9] In a substudy of the Gilead 102 trial comparing TDF/FTC plus either efavirenz or cobicistat-boosted elvitegravir, both as single tablet fixed-dose combination regimens, significant decreases were observed in several inflammatory markers in both arms including IL-6, soluble TNF receptor I and soluble CD163, but sCD14 and hsCRP decreased only in the elvitegravir group.[29] Thus, although the lack of change in IL-6 and hsCRP in our study is consistent with some prior studies,[30] it could also be related to the specific agents used. Further work is needed to elucidate the specific relationships between antiretroviral agents, viremia and individual inflammatory biomarkers.

The increase in sCD14 levels with undetectable viremia in our study was unexpected, given prior studies showing a decrease or plateau in this parameter with successful initiation of therapy.[9, 26–29] Soluble CD14 is a marker of lipopolysaccharide (LPS)-induced monocyte activation,[31] and as for the inflammatory and coagulation markers described above, higher plasma levels have been associated with earlier mortality.[32, 33] Only three prior studies have quantified changes in sCD14 in treatment-naïve adults initiating LPV/r, two of which one observed a decrease[34, 35] and one observed no change[27] over 12 months, 24 months and 72 weeks respectively. One cross-sectional study has suggested that protease inhibitors are associated with higher levels of monocyte activation including plasma sCD14 levels, but this was in the context of monotherapy.[36] Another study has suggested that sCD14 change on therapy may depend on baseline immunosuppression, with improvements seen only in those with moderate rather than severe immune depression,[35] but the population in our study had a reasonable median baseline CD4 count of 310 cells/mm^3^. Further work is needed to ascertain the reproducibility of our finding. Including additional markers of microbial translation such as circulating lipopolysaccharide in future studies could facilitate interpretation of the relationship between undetectable plasma viral load and sCD14; such markers could not be included in this study due to financial constraints.

Previous reports from the GARDEL trial have shown that the dual therapy regimen was virologically non-inferior to triple therapy over 96 weeks of follow-up, while also being associated with fewer toxicities and drug discontinuations.[6] Further, although dual therapy resulted in numerically greater cholesterol elevations than triple therapy, the distribution of lipids levels according to NCEP ATP goals for cardiovascular risk reduction was similar between groups.[37] While some of these findings may in part relate to the specific NRTI backbones selected in the triple therapy group (54% zidovudine/lamivudine in the trial overall), taken together, these results challenge the necessity of routinely including a second NRTI into traditional cART regimens.

Other trials have studied dual therapy regimens that combine lamivudine with a potent antiretroviral drug from another class. The ATLAS-M trial randomized 266 patients with plasma HIV RNA <50 copies/mL to either simplify their cART to atazanavir/ritonavir plus 3TC or continue on atazanavir/ritonavir plus two NRTIs. At 48 weeks, 89.5% of dual and 79.7% of triple therapy patients were free of treatment failure, for a difference (95% confidence interval) of 9.8% (1.2%, 18.4%), thus meeting pre-specified non-inferiority criteria as well as post-hoc criteria for superiority.[38] This difference was largely driven by a decrease in adverse events in the dual therapy arm, and as in the GARDEL trial, dual therapy patients had greater increases in lipid levels at week 48. Similar to our findings, no significant differences were seen in the changes between baseline and week 48 in CRP, IL-6, sCD14 and D-dimer between treatment arms.[39] The SALT trial investigated a similar approach to ATLAS-M, and again observed a non-inferior virologic response with dual (84%) versus triple (78%) therapy at 48 weeks, giving a difference of 6% (-5%, 16%).[40]

Three trials have combined lamivudine with the integrase inhibitor dolutegravir. In the exploratory PADDLE study, 18/20 treatment-naïve HIV-positive adults using this regimen had plasma HIV RNA <50 copies/mL at 48 weeks; of the other two, one committed suicide and the other later had HIV RNA <50 copies/mL at the end-of-study visit, without having changed regimens.[41] The GEMINI-1 and GEMINI-2 trials collectively randomized 1441 treatment-naïve HIV-positive adults to dolutegravir/3TC with or without tenofovir disoproxil fumarate, and found that the dual-therapy regimen was non-inferior to the triple therapy regimen at 48 weeks, with 91% versus 93% of participants having plasma HIV RNA <40 copies/mL, giving an adjusted treatment difference of -1.7% (-4.4%, 1.1%).[42] The LAMIDOL trial showed that 97% of 104 HIV-positive adults with plasma HIV RNA ≤50 copies/mL after at least 2 years of first-line triple therapy maintained virologic success on dolugravir/3TC at 48 weeks.[43]

Finally, once-daily oral dolutegravir/rilpivirine and 4-weekly injectable cabotegravir/rilpivirine have both been demonstrated to be viable dual therapy regimens for use as switch strategies virologically suppressed adults, in the SWORD and FLAIR trials, respectively.[44, 45] Key advantages of a dual therapy strategy include the potential for reduced toxicities and for reduced costs. However, with the exception of the ATLAS-M trial mentioned above, inflammatory marker levels in these dual therapy studies have not been reported.

Strengths of our study include the randomized design, variety of biomarkers studied, representing different inflammatory pathways, and large sample size relative to other published studies on inflammatory markers in treatment-naïve patients.

Our study also has limitations that warrant consideration. First, our analyses adjusted for a limited number of key covariates (age, sex, baseline CD4 count, and plasma HIV RNA) that are felt to impact on systemic inflammation in HIV, and the impact of unmeasured confounders on our results is unknown. For instance, we have previously shown that intercurrent infections and vaccinations can increase hsCRP levels by a clinically important magnitude of 2.244 ug/mL,[46] abnormal glucose metabolism is associated with immune activation in people living with HIV,[47] and smoking has been associated with increases in IL-6 and sCD14,[48] but data on these variables were unavailable. Second, while we selected a range of biomarkers relevant to HIV pathogenesis, it is possible that differences exist in inflammatory markers other than those studied herein. In particular, because only plasma was available for testing, we restricted our analyses to soluble biomarkers of inflammation, and could not directly assess T-cell and monocyte activation. Third, a large number of specimens yielded IL-6 levels below the assay LLOQ, and use of a more sensitive assay may have produced different results. Finally, use of an investigator-selected NRTI backbone in the triple therapy group, with the majority using the relatively older combination of AZT/3TC, may have introduced heterogeneity into inflammatory marker levels in that arm. However, the expected impact of different NRTI backbones on our results is unclear. For instance, while the HEAT trial reported smaller decreases in CRP with ABC/3TC than with TDF/FTC (-12% vs -20% geometric mean concentrations at 48 weeks),[49] the ACTG5202 trial reported 1.43 and 1.36-fold increases in CRP with ABC/3TC at 24 and 96 weeks respectively, but no change with TDF/FTC at these time points.[50] More data and larger cohorts are needed to understand how inflammatory biomarkers differ according to specific antiretroviral agents.

In summary, we found that dual therapy using lopinavir/ritonavir plus lamivudine was associated with similar inflammatory biomarker levels compared with triple therapy using lopinavir/ritonavir plus two nucleoside reverse transcriptase inhibitors in HIV-positive adults over 48 weeks. These findings support the further development of novel dual treatment regimens for HIV.
